# TCM‐Suite: A comprehensive and holistic platform for Traditional Chinese Medicine component identification and network pharmacology analysis

**DOI:** 10.1002/imt2.47

**Published:** 2022-08-15

**Authors:** Pengshuo Yang, Jidong Lang, Hongjun Li, Jinxiang Lu, Hanyang Lin, Geng Tian, Hong Bai, Jialiang Yang, Kang Ning

**Affiliations:** ^1^ Key Laboratory of Molecular Biophysics of the Ministry of Education, Hubei Key Laboratory of Bioinformatics and Molecular‐imaging, Center for Artificial Intelligence Biology, Department of Bioinformatics and Systems Biology College of Life Science and Technology, Huazhong University of Science and Technology Wuhan China; ^2^ Geneis Beijing Co., Ltd. Beijing China; ^3^ Department of sciences Qingdao Genesis Institute of Big Data Mining and Precision Qingdao Shandong China; ^4^ Academician Workstation Changsha Medical University Changsha China; ^5^ Sequenxe Biological Technology Co., Ltd. Xiamen China

**Keywords:** composition identification, database, holistic pipeline, network pharmacology, Traditional Chinese Medicine, webserver

## Abstract

DNA‐based biological ingredient identification and downstream pharmacology network analysis are commonly used in research for Traditional Chinese Medicine preparations (TCM formulas). Advancements in bioinformatics tools and the accumulation of related data have become driving forces for progress in this field. However, a lack of a platform integrating biological ingredient identification and downstream pharmacology network analysis hinders the deep understanding of TCM. In this study, we developed the TCM‐Suite platform composed of two sub‐databases, Holmes‐Suite and Watson‐Suite, for TCM biological ingredient identification and network pharmacology investigation, respectively, both are among the most complete: In the Holmes‐Suite, we collected and processed six types of marker gene sequences, accounting for 1,251,548 marker gene sequences. In the Watson‐Suite, we curated and integrated a massive number of entries from more than 10 public databases. Importantly, we developed a comprehensive pipeline to integrate TCM biological ingredient identification and downstream network pharmacology research, allowing users to simultaneously identify components of a TCM formula and analyze its potential pharmacology mechanism. Furthermore, we designed search engines and a user‐friendly platform to better search and visualize these rich resources. TCM‐Suite is a comprehensive and holistic platform for TCM‐based drug discovery and repurposing. TCM‐Suite website: http://TCM-Suite.AImicrobiome.cn.

## Highlights

- TCM‐Suite platform is composed of two sub‐databases, Holmes‐Suite and Watson‐Suite.
- Holmes‐Suite database covers six marker genes, resulting in 235,470 kinds of biological ingredients.
- Watson‐Suite retrieved massive data for mining the “herb‐compound‐protein‐disease” interpretation.
- A holistic pipeline was designed to connect Holmes and Watson, shown by a user‐friendly platform.

## Graphical abstract

TCM‐Suite platform is composed of two sub‐databases, Holmes‐Suite and Watson‐Suite. Holmes‐Suite database covers six marker genes, resulting in 235,470 kinds of biological ingredients. Watson‐Suite retrieved massive data for mining the “herb‐compound‐protein‐disease” interpretation. A holistic pipeline was designed to connect Holmes and Watson, showed by a user‐friendly platform.

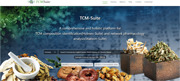

## INTRODUCTION

Traditional Chinese Medicine (TCM) is a branch of traditional medicine based on over 3500 years of Chinese medical practice encompassing various forms of herbal medicine [1, 2]. TCM preparation (i.e., formula) has considerable promise for treating various diseases and is a great resource for drug development [3–5]. With the advancement in bioinformatic tools and the accumulation of related data, TCM component (biological ingredient) identification based on advanced sequencing technology has become an emergent issue [6, 7]: For the accurate and efficient identification of TCM biological ingredients, it is crucial to generate a comprehensive and accurate DNA‐based marker sequence database, as well as to design efficient sequence search engines. Additionally, these TCM biological ingredient identification results could be linked to target proteins and molecular mechanisms of drug effects to enhance the understanding of the TCM formulas [8].

DNA‐based molecular marker genes (i.e., DNA barcodes) are efficient and accurate tools for identifying TCM biological ingredients [9, 10], especially in mixtures containing many species [11, 12]. DNA barcode is defined as a standardized short DNA sequence from a conservative region of a species' genome [10, 13], which can be used to delineate between taxonomic lineages [14, 15]. ITS2, *matK*, *trnH‐psbA*, *trnL*, *rpoc1*, *ycf1*, and other typical marker genes [16–18] are extensively used in TCM research. These DNA barcodes make it feasible and convenient to identify biological ingredients from TCM formula, which is a typical mixed system [19, 20]. Identification of TCM biological ingredients is essentially equivalent to identifying the taxonomy species belonging to various raw materials, depending on the precise identification of DNA barcodes of TCM biological ingredients. Using high‐throughput sequencing, the metagenomic approach has become one of the most important and effective approaches to deciphering the taxonomical profile of a mixture of biological ingredients based on marker genes [13, 21, 22], which could help to establish an accurate and efficient method for biological ingredient analysis of the TCM formula.

Typically, the TCM formula comprises of several herbs containing tens, hundreds, or even thousands of compounds [20, 21]. Consequently, TCM formulas represent a rich resource for clinical practice and drug development, while the identification of clear biological ingredients of TCM provides the basis for downstream pharmacological research. Each biological ingredient may contribute to the treatment of corresponding syndromes [23, 24]. Hence, after detecting the biological ingredients (i.e., herbs) of a TCM formula, investigation of the active compounds for each herb can effectively explore the herb's target genes and diseases [25, 26]. From a network perspective, network pharmacology may explain intricate relationships between biological systems of the active compounds (or small molecules), human proteins (or genes), and diseases [27–29]. When the herbs in the TCM formula are parsed, network pharmacology studies can decipher the role of a biological ingredient at the molecular level. Moreover, by examining the “compound‐protein‐disease” interaction network in medication processes, the potential effects of biological ingredients can be systematically assessed [30–32].

Recently, various databases have been developed for the analysis of TCM formulas, focusing on both the identification of biological ingredients and the inference of network pharmacology. For the TCM biological ingredient identification, databases like ITS2 Ribosomal RNA Database [33] and TCMBarcode Database [34] were constructed. For network pharmacology investigation, Super Natural II [35], TCM@Taiwan [36], ChEMBL [37], and TCMID [38] have been developed for the identification of formula‐herb and herb‐compound relationships for TCM formulas; STITCH [39] was constructed for compound‐protein association analysis, STRING [40] for protein–protein interaction analysis, and OMIM [41], and GAD [42] for protein‐disease association analysis. Finally, for a comprehensive understanding of TCM effects, toxicity [43], and side effect [43] databases have been developed for safety assessments.

Despite the remarkable success of these TCM‐related databases, the biggest bottleneck for the modernization and globalization of TCM research has not been overcome: what are the accurate component of a TCM formula, what are the special active compounds, and what are their targeted proteins in human disease? These questions remain largely unaddressed by any of the previously described TCM‐related databases. The limitations of current TCM‐related databases are as follows: (I) Currently, biological and chemical methods were far from complete and accurate. (II) For TCM biological ingredient identification, although many databases have been constructed for TCM network pharmacology analysis, the lack of integration and redundancy in multiple databases led to error‐prone, incomplete, and inefficient network pharmacology research. (III). Importantly, due to the current situation (e.g., lacking marker sequences for biological ingredients), a holistic pipeline is still lacking for TCM analysis, which includes identification of herbs of TCM formulas, mining their related active compounds and protein targets, and analysis of their associations with diseases.

In this study, we developed the TCM‐Suite database to facilitate TCM‐related information management, data‐driven hypothesis generation, and potential drug discovery. First, to construct a comprehensive database, a large number of entries covering different types of TCM‐related data were collected. For TCM biological ingredient identification, we collected sequences and related information for six marker genes (ITS2, *matK*, *trnH‐psbA*, *trnL*, *rpoc1*, and *ycf1*), resulting in the Holmes‐Suite sub‐database, containing 235,470 kinds of biological ingredients and 1,251,548 marker gene sequences. For downstream network pharmacology analysis, we retrieved large sets of public records, constructing the Watson‐Suite sub‐database, which includes 6,692 formulas, 7,322 herbs, 704,321 compounds, and 19,319 proteins, and 15,437 diseases, as well as corresponding correlations between different types of entries. Second, a holistic pipeline was developed to seamlessly integrate these two sub‐databases, covering the whole process from TCM biological ingredient identification to downstream network pharmacology investigation. Third, based on the enormous number of entries and holistic analysis pipeline, we designed a user‐friendly platform for users to explore and visualize these rich resources. We believe that TCM‐Suite could serve as a resource for TCM‐based drug discovery and repurposing, and that ongoing work on this integrated database and analytical platform will contribute to pharmaceutical research and clinical practice worldwide. The TCM‐Suite database website is available at http://TCM-Suite.AImicrobiome.cn.

## CONSTRUCTION AND CONTENT

### Data collection and processing for Holmes‐Suite

Raw sequences were obtained from the NCBI nucleotide database (https://www.ncbi.nlm.nih.gov/nucleotide/) in GenBank format in December 2020, using gene names as keywords (ITS2, *matK*, *trnH‐psbA*, *trnL*, *rpoc1*, and *ycf1*). We developed a TCM marker gene database and TCM ingredient identification system in the Holmes‐Suite database to satisfy these stringent requirements for TCM biological ingredient identification. The approach using machine learning to process different marker genes works as follows: First, for every type of marker gene, 1000 manually checked sequences representing clean and complete marker genes devoid of ambiguous base pairs and out‐of‐boundary sequences, as well‐annotated marker gene sequences, were selected. Second, a Hidden Markov Model (HMM) was trained using these well‐annotated marker gene sequences to identify the potential marker gene regions of these candidate sequences before these sequences could be included in our curated database. All downloaded sequences and sub‐sequences that did not fit the model were filtered out. Third, the sequences of positive entries not covered by the model were manually gathered. A detailed process workflow is exemplified by the ITS2 marker gene in the Holmes‐Suite database and provided on the help page (http://TCM-Suite.AImicrobiome.cn/help). Based on these high‐quality marker genes, a sequence‐based search engine with high accuracy and coverage was constructed.

### Data collection and processing for Watson‐Suite

Watson‐Suite was composed of six data fields: formula, herb, ingredient, compound, protein, and disease. In the Watson database, different data fields were linked by direct correlations collected and curated from the existing database. Based on the data in existing databases, the direct correlations include formula‐herb correlations, herb‐ingredient, ingredient‐compound, compound‐protein, and protein‐disease [44, 45]. the other correlations among these six data were defined as indirect correlations, calculated based on the network pharmacology.

For these collected data from different databases, data integration and filtration were conducted, followed by manual annotations and corrections. First, to improve the compatibility of data, we converted all of them into a standard format. Second, based on these integrated data, each entry was manually checked and tagged with a unique label, and then duplicate records were removed. The detailed process procedure was supplied on our website(http://TCM-Suite.AImicrobiome.cn/help).

### Interconnection between entries in Holmes‐Suite and Watson‐Suite

**Figure 1:**
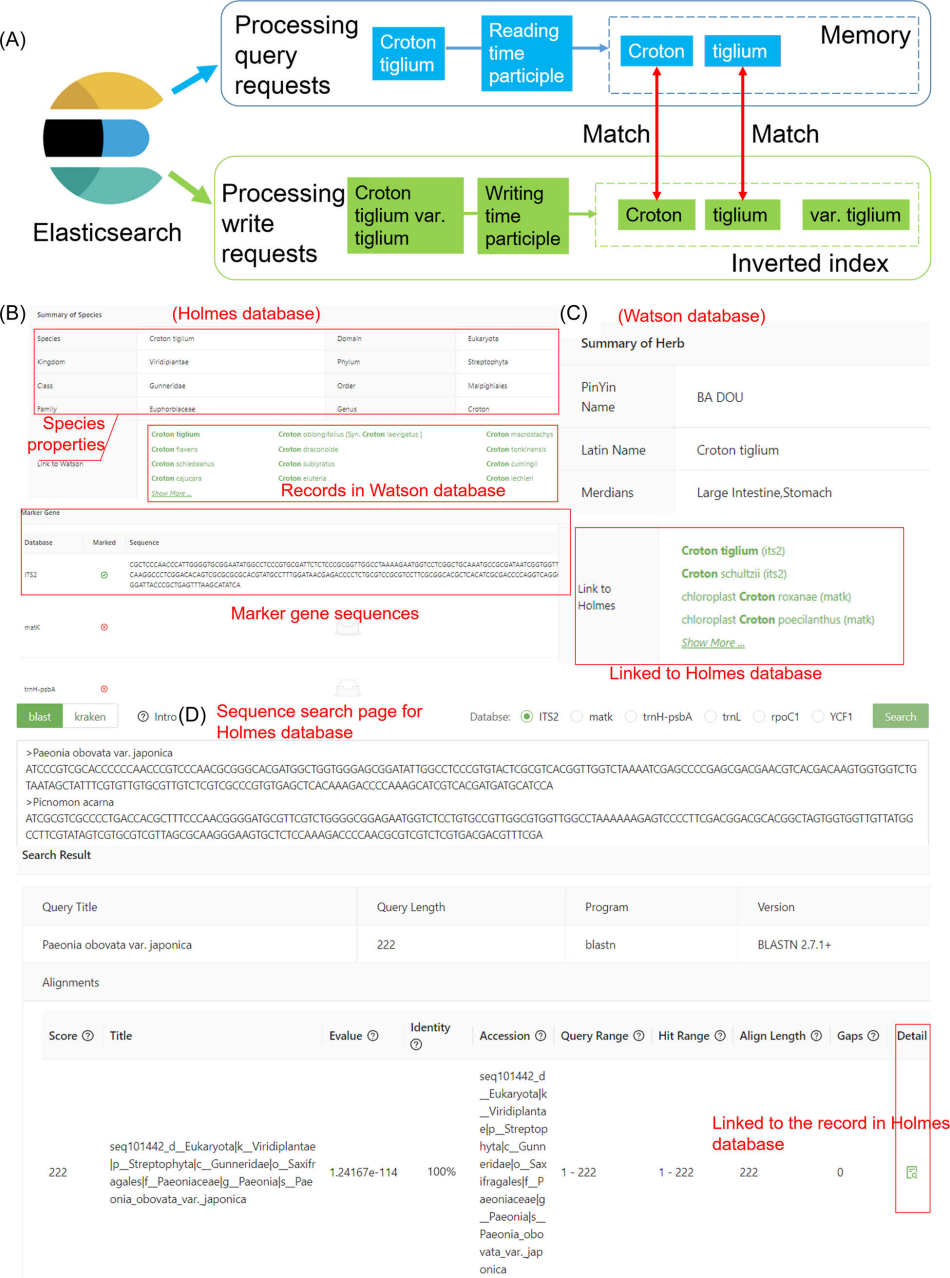
Interconnection of Holmes‐Suite and Watson‐Suite sub‐databases. (A) Fuzzy matching of biological ingredients for Holmes‐Suite and Watson‐Suite connectivity. The “species” field in Holmes‐Suite was linked to the “herb” field in Watson‐Suite via Elastic search. (B) Entry‐specific information from the Holmes‐Suite sub‐database. The details of each entry contained the taxonomical classification. The frame of “Link to Watson” provided the hyperlink for corresponding network pharmacology analysis in Watson‐Suite. (C) Detailed herbal information page for Watson‐Suite database. The species name and drug‐related properties for this entry were provided. Similarly, the hyperlink for the species in Holmes‐Suite was provided. (D) The sequence search result in the Holmes‐Suite database also supplied the hyperlink to the record in Holmes‐Suite. Users could conveniently obtain the taxonomical information of this search result and the corresponding network pharmacology information.

This interconnection was established by connecting the “species” field in Holmes‐Suite and the “herb” field in Watson‐Suite, as both fields indicated the Latin name of the biological ingredient of TCM‐related herbs (Figure 1A). Fuzzy matching and Elastic search (https://www.elastic.co) were used to match these two fields. For the matched herbs, we integrated the detailed information of the two sub‐databases on the website for the TCM biological ingredient in Holmes‐Suite (Figure 1B) or determination of the target herb in Watson‐Suite (Figure 1C), while providing a hyperlink to the corresponding database. Similarly, this interconnection was also uncovered using the Holmes‐Suite search module (Figure 1D).

### Implementation of TCM‐Suite

The TCM‐Suite website was developed using the Python‐Flask, Nginx, and React JavaScript frameworks and is compatible with most major browsers (tested with Chrome, Edge, Firefox, and Safari) under different operating systems. TCM‐Suite database supports 10 people to search the database and 30 people to browse online at the same time. TCM‐Suite stores its data in a PostgreSQL database. TCM‐Suite is dedicated to providing a user‐friendly web interface for users to browse, search, visualize, and download data. TCM‐Suite is freely accessible at http://TCM-Suite.AImicrobiome.cn without any registration.

### Data resources and database construction

To establish a comprehensive database, we collected and integrated data from multiple sources for the construction of the TCM‐Suite database: To incorporate the marker gene sequences for identifying the TCM biological ingredient, the Holmes‐Suite sub‐database was constructed (http://TCM-Suite.AImicrobiome.cn/holmes/browse). For downstream network pharmacology analysis, the Watson‐Suite sub‐database was constructed (http://TCM-Suite.AImicrobiome.cn/watson/browse). Watson‐Suite contains up to 6,692 formulas, 65,231 formula‐herb associations, 7,322 herbs, 54,868 herb‐compound associations, 704,321 compounds, 4,350,151 protein‐compound associations, 19,319 proteins, and 814,250 gene target sites for proteins, 160,095 protein‐disease related records, 15,437 diseases, and 160,169 side effect records. The databases from which these entries were collected are summarized in Supporting Information: Table 1.

**Figure 2:**
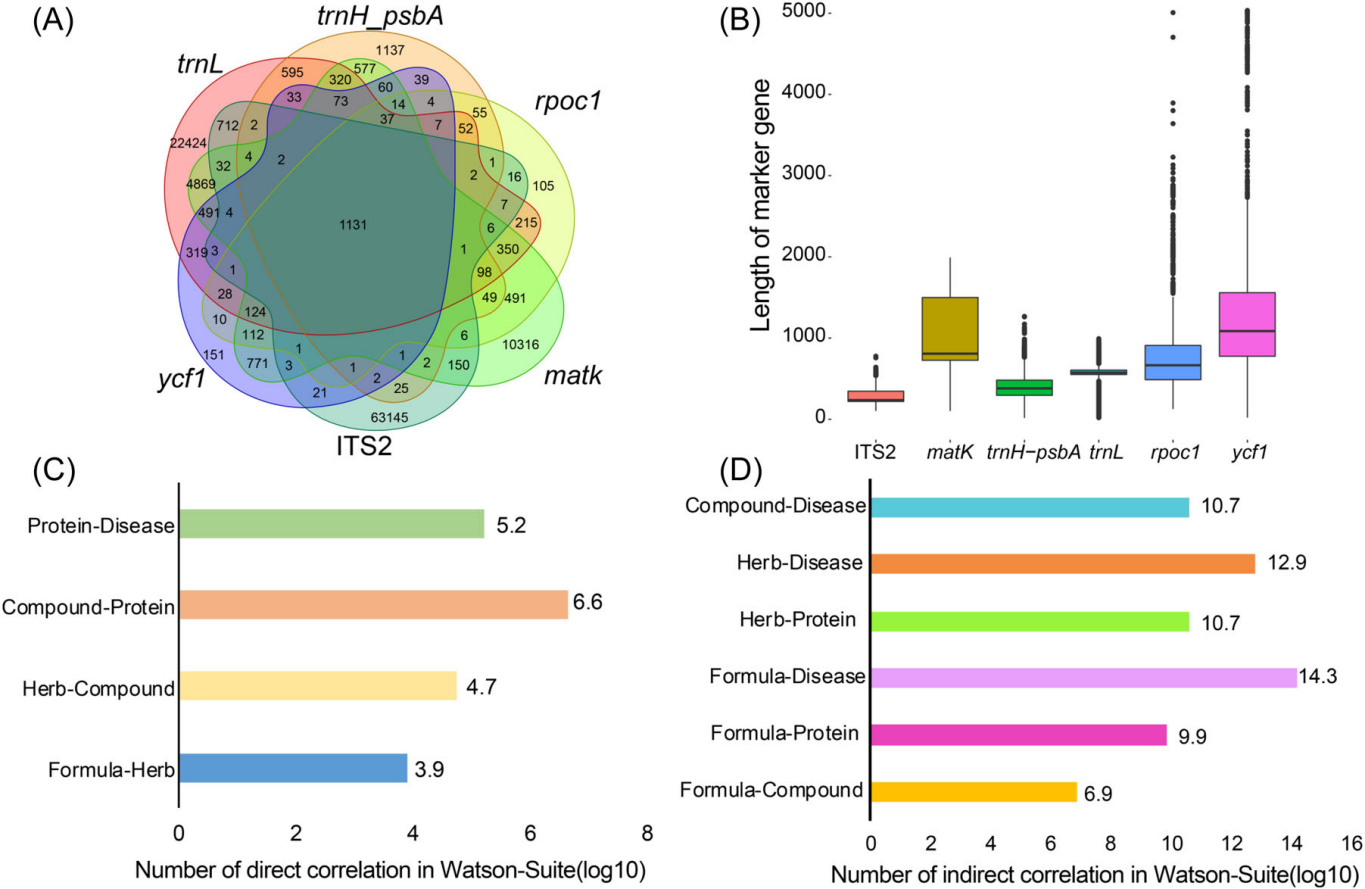
Statistical results for the Holmes‐Suite and Watson‐Suite sub‐databases. (A) Distribution of species across six marker genes. The Venn diagram illustrates the number of species grouped by six marker gene databases. Six types of marker gene share 1131 common species. (B) Sequence length distribution for six marker genes. For each type of marker gene, length distribution was calculated and labeled in different colors. (C) Direct correlations for six categorizations of pharmacology networks in the Watson‐Suite sub‐database. Direct correlations were collected and curated from the existing database. Bar plots show the total number of direct associations for five types of direct associations. (D) Indirect correlations for five categorizations of pharmacology networks in the Watson‐Suite database. Indirect correlations were deduced by network pharmacology analysis. Bar plots show the total number of indirect associations for five direct associations.

Detailed statistics of the data in the Holmes and Watson databases were depicted in Figure 2. In the Holmes database, the species distribution for six marker genes was presented (Figure 2A). According to this species distribution, the six marker genes have distinct species distributions, although they nevertheless share 1131 species entries. The high quality of these marker genes is reflected by a long average length based on the length distribution (Figure 2). In the Watson database, the characters of pharmacology networks for different types of entries are reflected by direct correlations collected and curated from the existing database (Figure 2C) and indirect correlations deduced by network pharmacology analysis (Figure 2D). Compared to previous databases, our database has improved both the number of marker genes and the complexity of the pharmacology network [44, 45].

### Evaluation of the Holmes‐Suite database

**Figure 3:**
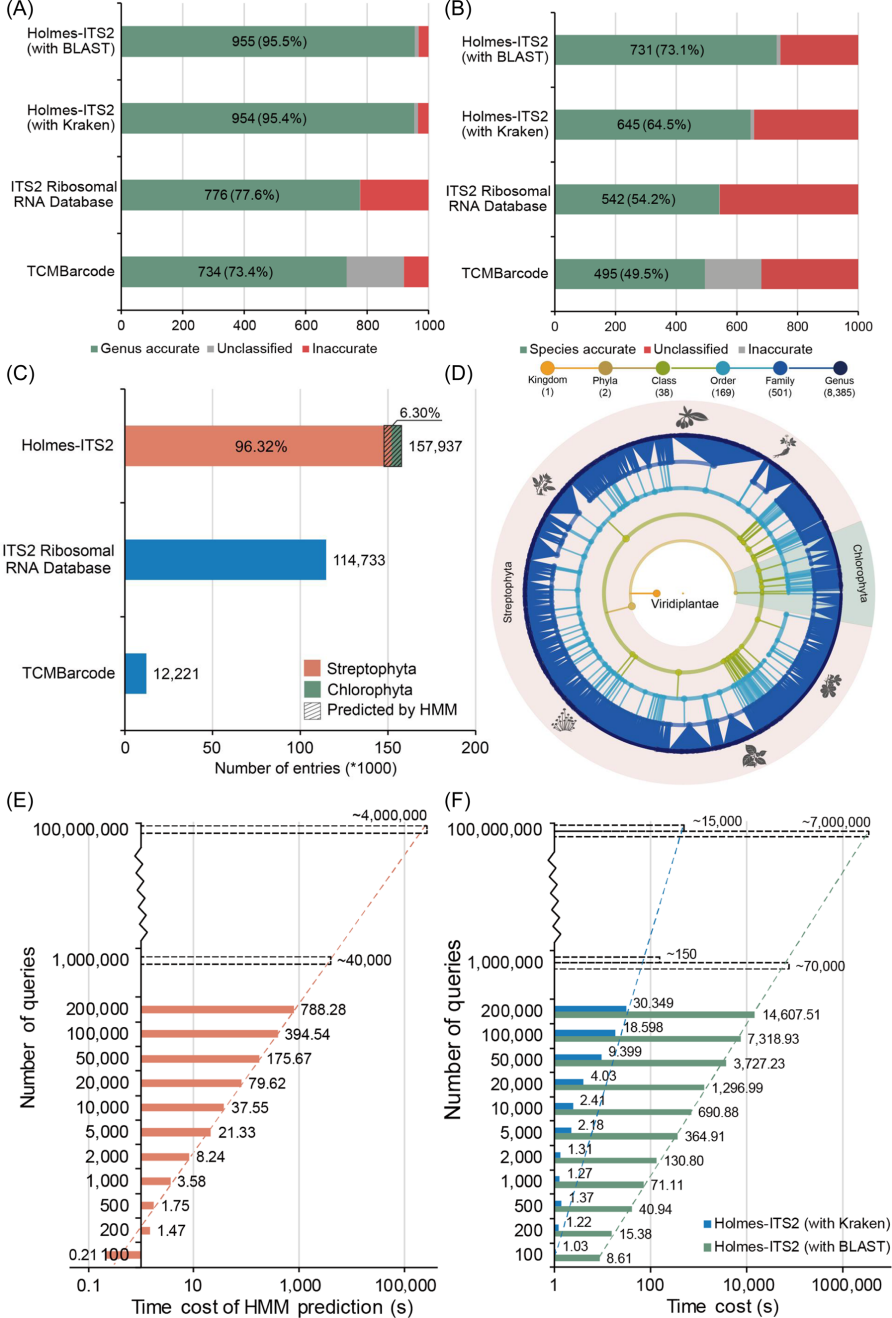
Comparison of identification accuracy using ITS2 across the three databases Holmes‐Suite, ITS2 Ribosomal RNA, and TCMBarcode databases. (A) Genus‐level and (B) species‐level sensitivities for the three databases. For each of the horizontal histograms, the number inside each bar shows the number and rate of sequences correctly identified at the target taxonomy level. (C) The number of entries (DNA‐based marker sequences) contained in each database (blue) and data component of Holmes‐ITS2 (ITS2 part of the Holmes‐Suite database). Note that only plant species were used here for comparison. (D) Holmes‐Suite covers a relatively complete phylogenetic tree for species identifiable by ITS2. From the inside to outside of the taxonomy hierarchical tree, each node represents a taxon with a distinct level of taxonomy, such as kingdom, phylum, or class. Each connection represents an affiliation of the outer node to the inner node. (E) HMM prediction for training the ITS2 sequence edge model and (F) identification with BLAST and Kraken search engines. Solid horizontal bars represent the time cost of the classification procedure for a different number of queries. Trend lines of the time cost of different procedures are illustrated by dashed lines. The classification time cost of different steps showed a linear increase; hence, time costs under one million and one hundred million queries are projected values indicated by horizontal dashed line bars.

The requirements of high‐throughput analysis of TCM biological ingredients have resulted in an elevated standard for accuracy (high sensitivity required), completeness (cover as many species as possible), as well as efficiency (processing batch and bulk data quickly) of identification. To evaluate the accuracy, completeness, and efficiency of the Holmes‐Suite database, we compared it to two existing databases: ITS2 Ribosomal RNA Database (http://its2.bioapps.biozentrum.uni-wuerzburg.de/) and TCM‐Barcode Database (http://www.tcmbarcode.cn/en/), using marker gene ITS2 (1000 ITS2 raw entries were randomly selected from NCBI as queries, restricted to the plant ITS2), evaluated both on genus and species levels. The data was downloaded in December 2020. The comparison result illustrated that Holmes‐Suite performs better in searching accuracy, searching efficiency, and data coverage compared to the other two databases (Figure 3).

First, the search accuracy for the three databases was evaluated and compared, revealing that Holmes‐Suite outperforms the other two. For accuracy benchmark on genus level, the Holmes‐Suite database using BLAST as the search engine achieved the highest score among the three databases (Figure 3A), with a sensitivity of up to 95.5%; whereas the Holmes‐Suite database using Kraken as search engine reached 95.4% sensitivity. The sensitivity of the Holmes‐Suite database with both search engines was greater than that of TCMBarcode (73.4%) and the ITS2 Ribosomal RNA database (77.6%). For the species‐level accuracy benchmark (Figure 3B), the Holmes‐Suite database using BLAST as a search engine achieved the highest classification sensitivity (73.1%) across all three databases, demonstrating a substantial advantage (Holmes with Kraken search engine: 64.5%; TCMBarcode:5 4.2%; ITS2 Ribosomal RNA database: 49.5%). Consequently, Holmes‐Suite showed the best performance in classification accuracy among the three databases. Although our findings are based on ITS2 sequences as query sequences, we believe that when processed with the same pipeline, the results on the other six marker genes would also be very accurate.

Second, the database completeness for the three databases was evaluated and compared (Figure 3C,D). With an order of magnitude larger database of Holmes‐ITS2 (ITS2 part of the Holmes‐Suite database) than the ITS2 Ribosomal RNA database and the TCMBarcode database (Supporting Information: Table 2, Figure 3C), the TCM‐Suite would cover a rather comprehensive phylogenetic tree for species identifiable by ITS2 (Figure 3D). Interestingly, given that Holmes‐Suite was far bigger than other databases, its higher sensitivity may indicate the superior quality of the content of the Holmes‐Suite database. However, arguably, bigger databases are not always associated with the cost of slower database queries. This is not the case for the Holmes‐Suite database.

Third, the efficiency was evaluated by the classification speed of two main procedures (Figure 3E,F). The time cost analysis on the HMM model (a trained model to trim off the ambiguous boundary regions of the download ITS2 sequences for higher accuracy, see Materials and Methods) (Figure 3E) processing and searching by BLAST (Figure 3F) have shown that these costs have linear relationships with the increase in query number; on average, the speed of the two procedures were 250 queries and 15 queries per second, respectively, based on tests using our web server. It is noteworthy that the time cost of classification by Kraken, which did not significantly rise with the number of queries tested, is an average classification speed of more than 6,000 sequences per second. During the process of identification of a small amount of data, fluctuation in time cost resulted because of a significant difference from the maximum identification speed of Kraken.

### Evaluation of Watson‐Suite database

To evaluate the accuracy and efficiency of the Watson‐Suite database, 125 compounds from the herb *Panax ginseng* (i.e. ginseng) were used as a search query and compared to the TCMID database [46] (Supporting Information: Figure 1). For this herb, 60 compounds were retrieved from the Watson‐Suite database, however, the TCMID database only returned 44 compounds (Supporting Information: Figure 1A). Then, we evaluated the pharmacology network associated with ginseng retrieved from the Watson‐Suite database: To identify the most reliable correlations, we selected 10 genes for each of the 21 compounds, ranked by the combined score [47, 48], yielding a collection of 152 genes and 483 pairs of gene interactions for the 152 genes. By targeting some of these 152 genes, ginseng could exert effects on a variety of diseases, such as protopine, sleep disorder, atherosclerosis, diabetes, Alzheimer's disease, hypertension, cardiac dysrhythmias, insulin resistance, pyresis, and depression. Based on the correlations with a large number of genes and diseases (Supporting Information: Figure 1B), these 21 compounds were related to the biological traits of the ginseng, demonstrating the pharmacological activity of ginseng similar to previous studies [49, 50].

To validate the “herb‐compound‐protein‐disease” network generated for ginseng, we investigated the main active compounds of ginseng and discovered that the ginsenoside Rg1‐related genes, i.e., TNF, are related to diabetes, Alzheimer's disease, hypertension, and atherosclerosis, which was consistent with previous studies [51, 52]. In addition, a ginsenoside Rb1‐related gene, AKT1, was found to be associated with diabetes. Furthermore, the genes related to other compounds of ginseng identified from the TCM‐Suite, such as ADA, ADK, ADORA2A, and GCG, have been previously reported to be associated with sleep disorders [53, 54] and diabetes [55].

To further evaluate the efficiency of the Watson‐Suite database searches, 100 FDA‐approved drugs were selected randomly from DrugBank [56] as a benchmark data set. For all of these 100 drugs, the average searching time was 0.648 s and 0.617 s for the first and second queries, respectively (Supporting Information: Figure 1C). As the TCM‐Suite could save the result of the first search in a cache, the second query consumed less time. Furthermore, we used varying quantities of compounds to search in our database (Supporting Information: Figure 1D). The result showed that retrieval efficiency grows linearly as the quantity of compounds increases.

## UTILITY AND DISCUSSION

### A holistic pipeline for TCM analysis

To facilitate more in‐depth mining of the massive amount of information on natural products and the clinical application of TCM, we realized the interconnection of the marker gene database (Holmes‐Suite) and downstream network pharmacology analysis (Watson‐Suite). Based on the machine learning method, 153,016 species entries (65% of all 235,470 species entries) in TCM‐Suite could be matched to the “herb” field in Watson‐Suite. For the matched herbs, we integrated the detailed information from the two sub‐databases. Consequently, this holistic pipeline allowed users to search for a TCM‐source sequence, for which TCM‐Suite would return the matched entries in the Holmes‐Suite database utilizing the sequence‐based search engine. Additionally, as all the data were interconnected, TCM‐Suite would also return the related network pharmacology information. And vice versa, the user could query the interesting entry in Watson‐Suite and would return all the related entries in the Watson‐Suite database by network pharmacology analysis. TCM‐Suite would also return the related marker gene information in Holmes‐Suite. This integrated pipeline highlights the explainability of our database, hence broadening and deepening TCM‐related data mining.

### Database features and web interface

**Figure 4:**
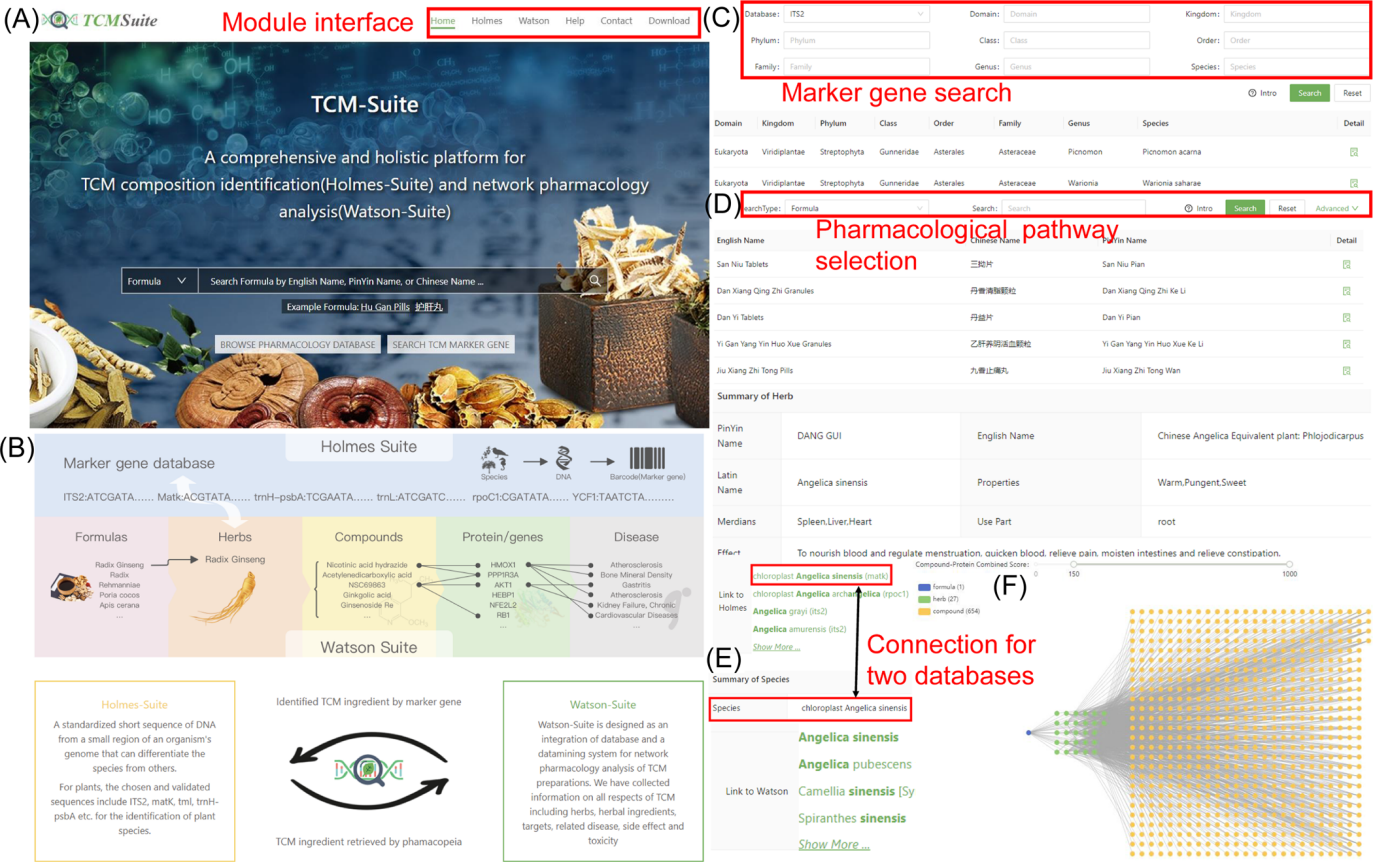
Screenshots of TCM‐Suite databases interface. (A) The homepage of TCM‐Suite. In the top right corner, hyperlinks to all modules are provided. (B) The browsing page of marker gene ITS2 for Holmes‐Suite. Detailed taxonomical classification is shown, along with a detailed description linked to Wikipedia. (C) The front page of Holmes‐Suite. The browse page of marker gene entry in Holmes‐Suite consisted of two parts: taxonomical information and a link to Watson‐Suite for mining downstream network pharmacology information. (D) The detailed information page of an herb for Watson‐Suite. All the records for this herb are listed at the top of the page, followed by the link to marker gene sequences in the Holmes‐Suite database. The pharmacology network generated for the selected herb in the Watson‐Suite database is shown. Information was displayed when the cursor was pointed to the corresponding area. Finally, the detailed information and corresponding link were provided under the network and organized by category of entry.

The TCM‐Suite database provides an easy‐to‐use interface for users to browse data and search related entries in the database and mine the related information. The front page and data browsing pages for TCM‐Suite are shown in Figure 4: On the homepage of TCM‐Suite (Figure 4A), the key functions of the TCM‐Suite were provided: on the top right, links to all modules were shown. Additionally, we provided an interface for users to directly search all the database entries. The search interface was followed by basic database statistics, and the conceptions of TCM‐Suite were also described (Figure 4B), providing the logic principles behind our database. On the Holmes‐Suite sub‐database, the browse page (Figure 4C) listed all the marker gene entries after the selection of the marker gene. The column labeled “Detail” provided the hyperlink to the classification information for each entry and the link to Watson‐Suite, which provided the corresponding network pharmacology information. The browse page of Watson‐Suite (Figure 4D) displayed all the entries in the database after selection of the category of network pharmacology. For each entry in the Watson‐Suite database, the associated data obtained from other categorizations were also listed (Figure 4F). To comprehensively and intuitively display the complex correlations among different categorizations of entries, we designed a visualization interface using Cytoscape Web (http://cytoscapeweb.cytoscape.org/) to show the associations in the pharmacology network. If the detailed information contains the category of “herb” (i.e., if the marker sequence is accessible), a link to the corresponding records in Holmes‐Suite was provided on the “detail” page (Figure 4E).

### Network pharmacology investigation for COVID‐19

The coronavirus disease 2019 (COVID‐19) has garnered international attention [57, 58]. Based on a large number of clinical studies, several TCM formulas were proven to be effective in the treatment of COVID‐19 and were included in the guidelines on COVID‐19 diagnosis and treatment [59, 60]. As a powerful platform for integrating TCM resources, the TCM‐Suite platform also allows the investigation of COVID‐19‐related pharmacology networks for the discovery of potential gene targets and therapeutic drugs.

Based on previous studies, five candidate genes were selected as the targets for treating COVID‐19 were proposed [61, 62]. For these candidate genes, we constructed the “herb‐protein‐disease” networks using our database. For the five query genes (ACE2, SLC6A20, SIT1, CCR9, and CXCR6), we identified compounds (12, 23, 1, 2, and 14, respectively) and herbs (58, 128, 2, 8, and 54, respectively) based on the entries in our database. Among these five genes, most records in our database were identified as being associated with genes SLC6A20 and ACE2, which was also observed in previous studies [61, 62]. Further investigations into these potential target genes indicated that most of the effects of the 152 herbs could act through “clear heat and detoxify” and “facilitate the flow of the lung and relieves toxicity,” consistent with previous studies [63, 64].

We also investigated COVID‐19 clinical symptoms to construct pharmacological networks. Based on the COVID‐19 clinical symptoms identified in previous studies [65], we conducted a network pharmacological survey (Supporting Information: Table 3). Pneumonia is an important symptom of COVID‐19 infection [66, 67], and interestingly, many records in the TCM‐Suite database were associated with “pneumonia.”

We combined these pharmacological networks and obtained a target candidate gene cluster and potential therapeutic drugs for COVID‐19. The common target genes and biological ingredients in these networks (in over five pharmacological networks) were calculated, resulting in a total of 15 drug targets, 52 compounds, and 86 biological ingredients. Many of these target genes have also been identified in previous studies, such as ACE2, SLC6A20, SIT1, CCR9, and CXCR6 [68–71]. Many of these 86 biological ingredients were identified as important components of clinically effective TCM to cure COVID‐19 [69]. For example, the biological ingredients *Lonicera japonica*, *Scutellaria baicalensis*, and *Forsythia suspensa* are the active ingredients of Shuanghuanglian; *Forsythia suspensa*, *Ephedra sinica*, *Lonicera japonica*, *Isatis indigotica*, *Mentha haplocalyx*, and *Dryopteris crassirhizoma* are the main components of the Lianhua Qingwen capsule. All these TCM formulas were identified as effective for COVID‐19 treatment [69–71]. In this case study, the TCM‐Suite platform showed immense potential for the evaluation of TCM's effectiveness on diseases.

## CONCLUSION

Existing databases for TCM biological ingredient identification and network pharmacology inference have limited in search accuracy, database completeness, and search speed. In developing the TCM‐Suite, we aimed to consolidate and combine existing database resources to facilitate the seamless integration of TCM biological ingredient identification (Holmes‐Suite) and downstream network pharmacological analysis (Watson‐Suite) into a holistic analysis platform. For TCM biological ingredient identification, we developed a highly accurate, comprehensive, and efficient identification system Holmes‐Suite, which is user‐friendly and highly interactive, and empowered by machine learning techniques such as HMM for entry matches and sequence searches. The collection of data from six marker genes (ITS2, *matK*, *trnH‐psbA*, *trnL*, *rpoc1*, and *ycf1*) resulted in the Holmes‐Suite sub‐database, covering 235,470 kinds of biological ingredients and 1,251,548 marker gene sequences. This would boost the usage of Holmes‐Suite and its value for effective biological ingredient identification for TCM formulas. For downstream TCM‐related network pharmacology analysis, Watson‐Suite enables deeper mining of the pipeline for “herb‐compound‐protein‐disease” interpretation. The Watson‐Suite sub‐database covers 6,692 formulas, 7,322 herbs, 704,321 compounds, 19,319 proteins, and 15,437 diseases, and corresponding network pharmacology entries. Watson‐Suite can establish a comprehensive view of network pharmacology by integrating existing data.

TCM‐Suite achieved a smooth interconnection between the two sub‐databases, Holmes‐Suite and Watson‐Suite. Our platform has great potential for TCM‐based drug discovery and repurposing, which is reflected in our benchmark work. In the benchmark of Holmes‐Suite, 1,000 ITS2 raw entries randomly selected from NCBI served as test data set queries found that 552 (55.2%) of them could be retrieved in Watson‐Suite for network pharmacology analysis. Similar interconnections were observed in the benchmark of Watson‐Suite. These results demonstrated a strong connection between Holmes‐Suite and Watson‐Suite databases, but also revealed insufficient biological ingredient information, as well as network pharmacology information, for TCM formulas.

Additionally, the high speed of TCM biological ingredient identification and network pharmacology search empowers TCM‐Suite for effective application. Compared to other databases, the query speed of Holmes‐Suite is faster and a linear relationship was detected between the time required for the query and the number of queries. Both results reflect the high efficiency of the search engine inside the Holmes‐Suite sub‐database. Meanwhile, Watson‐Suite also works rapidly: when querying 100 drugs, the reaction time of the database was almost 0.6 s. Moreover, with a user‐friendly interface, the TCM‐Suite could be used easily for many pharmacology applications. Taken together, TCM‐Suite is a rich resource for TCM‐based drug discovery and repurposing and could contribute to both fundamental and clinical research.

TCM research, especially that related to molecular biology, is becoming popular, and huge amounts of data have been accumulated. TCM‐Suite is planned to be updated bi‐annually to reflect the latest advancements in biological ingredient identification and network pharmacology. Additionally, more advanced artificial intelligence techniques, such as deep learning for more accurate TCM ingredient identification model building (not limited to biological ingredient only), as well as better data mining techniques, such as network motif discovery for network pharmacology examination, may be applied. We believe continuous efforts in this integrated database and analytical platform will contribute to pharmaceutical research and clinical practice worldwide.

## AUTHOR CONTRIBUTIONS

Hong Bai, Pengshuo Yang, Hongjun Li and Kang Ning conceived and proposed the idea, and designed the study. Pengshuo Yang, Jidong Lang, and Hongjun Li performed the experiments. Hong Bai, Pengshuo Yang, Hongjun Li, Jidong Lang, Geng Tian, and Kang Ning analyzed the data. Hong Bai, Pengshuo Yang, Hanyang Lin, Jingxiang Lu, Jialiang Yang and Kang Ning. contributed to editing and proofreading of the manuscript. All authors read and approved the final manuscript.

## CONFLICT OF INTEREST

Jidong Lang and Jialiang Yang are employed by Genesis Beijing Co. Ltd. The remaining authors declare no conflict of interest.

## Supporting information

Supporting information.

## Data Availability

The TCM‐Suite database website is available at http://TCM-Suite.AImicrobiome.cn/. Supplementary materials (figures, tables, scripts, graphical abstract, slides, videos, Chinese translated version and update materials) may be found in the online DOI or iMeta Science http://www.imeta.science/.
