# Supplementary material for: TCM‐Suite: A comprehensive and holistic platform for Traditional Chinese Medicine component identification and network pharmacology analysis
Source: Imeta. 2022 Aug 15;1(4):e47. doi: 10.1002/imt2.47 (PMC10989960; doi:10.1002/imt2.47)
Supplement: Supplementary file 1 — Supporting information. [file IMT2-1-e47-s001.docx]

**Supplementary file for “TCM-Suite: A comprehensive and holistic platform for TCM component identification and network pharmacology analysis”**

Pengshuo Yang^1,#^, Jidong Lang^2,3,4,#^, Hongjun Li^1^, Jinxiang Lu^1^, Hanyang Lin^5^, Geng Tian^2,3^, Hong Bai^1,*^, Jialiang Yang^2,3,4,*^, Kang Ning^1,*^

^1^ Key Laboratory of Molecular Biophysics of the Ministry of Education, College of Life Science and Technology, Huazhong University of Science and Technology, Wuhan, Hubei 430074, China

^2^ Geneis Beijing Co., Ltd., Beijing 100102, China

^3^ Qingdao Geneis Institute of Big Data Mining and Precision Medicine, Qingdao, 266000, China

^4^ Academician Workstation, Changsha Medical University, Changsha 410219, China

^5^ Sequenxe Biological Technology Co., Ltd., Xiamen 361000, China

^#^ These authors contribute equally

* To whom correspondence should be addressed. Email: ningkang@hust.edu.cn; yangjl@geneis.cn

**Content**

[**Supplementary Figures** 3](#_Toc109659052)

[**Figure 1. Accuracy and efficiency analysis for the Watson-Suite sub-database.**. 3](#_Toc109659053)

[**Supplementary Tables** 4](#_Toc109659054)

[**Table 1. Data sources for the TCM-Suite and detailed information of different databases.** 4](#_Toc109659055)

[**Table 2. Databases used for comparison of biological ingredient of TCM.** 6](#_Toc109659056)

[**Table 3. Results for searching five COVID-19 clinical symptoms based on network pharmacology analysis.** 7](#_Toc109659057)

**Supplementary Figures**


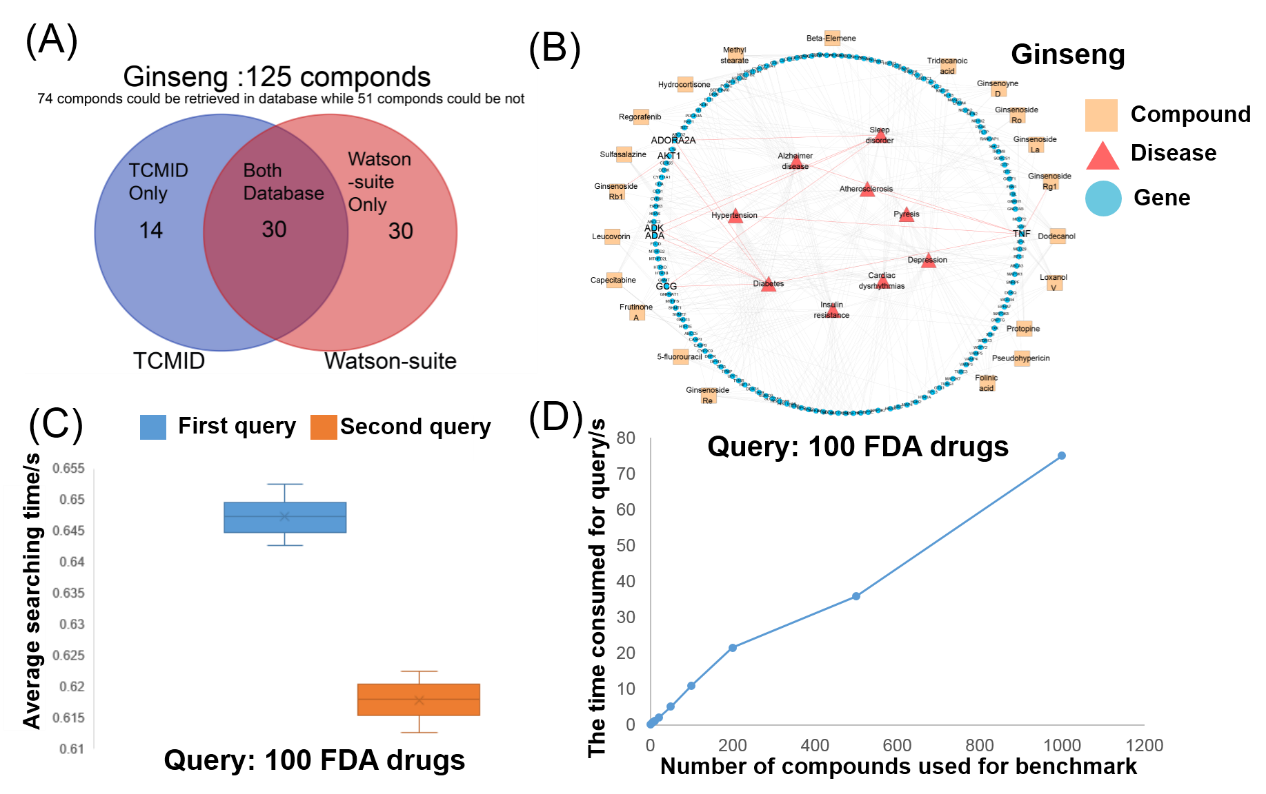


**Figure 1. Accuracy and efficiency analysis for the Watson-Suite sub-database.** (A) Comparison of the Watson-Suite and TCMID by evaluating how many compounds of “ginseng” could be retrieved. The number in the circle represents the amount of compounds that could be retrieved with the corresponding hits in the databases. (B) Pharmacology network generated for “ginseng” in the Watson-Suite database. The related compounds, protein, and corresponding disease are displayed hierarchically. Important compounds/disease/gene and their connections are highlighted. (C) Average time spent for the first (0.648 s) and second (0.617s) queries for 100 FDA-approved drugs. The result was calculated by random queries. (D) Search time for querying Watson-Suite varied with the amount of compounds.

**Supplementary Tables**

**Table 1. Data sources for the TCM-Suite and detailed information of different databases.**

| **Data entity** | **Data source** | **# of entries** | **Description** | **Websites** |
| --- | --- | --- | --- | --- |
| **Barcode genes (Holmes-Suite)** | NCBI | 157,937 | marker gene ITS2 | https://www.ncbi.nlm.nih.gov/nucleotide/ |
|  | NCBI | 24,267 | marker gene *matK* | https://www.ncbi.nlm.nih.gov/nucleotide/ |
|  | NCBI | 5,418 | marker gene *trnH*-psbA | https://www.ncbi.nlm.nih.gov/nucleotide/ |
|  | NCBI | 39,643 | marker gene *trnL* | https://www.ncbi.nlm.nih.gov/nucleotide/ |
|  | NCBI | 4,086 | marker gene *rpoc1* | https://www.ncbi.nlm.nih.gov/nucleotide/ |
|  | NCBI | 4,119 | marker gene *ycf1* | https://www.ncbi.nlm.nih.gov/nucleotide/ |
| **Formula (Watson-Suite)** | TCM-ID | 6,692 | Formula and corresponding  herb information | http://bidd.group/TCMID/ |
| **Herb**  **(Watson-Suite)** | TCMID | 57,249 | Herb information | http://www.megabionet.org/tcmid/ |
|  | TCMSP | 29,384 | Herb information | http://tcmspw.com/tcmsp.php |
| **Compounds**  **(Watson-Suite)** | STITCH | 82,841,024 | Compound-CID number links | http://stitch.embl.de/ |
|  | TOXNET | 103 | Compound’s toxicity | https://toxnet.nlm.nih.gov/ |
|  | TCMSP | 12,144 | Compound | http://tcmspw.com/tcmsp.php |
|  | SIDER | 163,221 | Compound’s side effect | http://sideeffects.embl.de/ |
| **Proteins**  **(Watson-Suite)** | STITCH | 4,523,609 | CID number-protein links | http://stitch.embl.de/ |
|  | STRING | 4,274,001 | Protein interactions | http://www.string-db.org/ |
|  | OMIM | 2,449,433 | Proteins and corresponding aliases | http://omim.org/ |
|  | OMIM | 15,591 | Approved gene and MIM number | http://omim.org/ |
| **Diseases (Watson-Suite)** | OMIM | 7,086 | MIM number-disease links | http://omim.org/ |
|  | TCMSP | 837 | Protein-disease associations | http://tcmspw.com/tcmsp.php |
|  | GAD | 167,130 | Protein-disease associations | https://geneticassociationdb.nih.gov/ |

**Table 2. Databases used for comparison of biological ingredient of TCM.**

| **Existing Database** | **Number of sequences** | **Latest update time** | **Website** |
| --- | --- | --- | --- |
| **ITS2 Ribosomal RNA Database** | 113,364 | 2015-08-07 | http://its2.bioapps.biozentrum.uni-wuerzburg.de/ |
| **TCMBarcode**  **Database** | 452,517 | 2018-05-06 | http://www.tcmbarcode.cn/en/ |
| **Holmes-Suite**  **Database** | 1,251,548 | 2021-07-27 | http://TCM-Suite.AImicrobiome.cn |

**Table 3. Results for searching five COVID-19 clinical symptoms based on network pharmacology analysis.**

| **Disease** | **# Gene target** | **# Compound** | **# Herb** |
| --- | --- | --- | --- |
| SARS-coronavirus | 3 | 54 | 156 |
| Pneumonia | 10 | 67 | 138 |
| Typhoid fever | 8 | 60 | 215 |
| Cough | 5 | 46 | 215 |
| Dyspnea | 1 | 13 | 100 |
| Diarrhea | 12 | 25 | 125 |
